# Divergent Effects of Palm Tocotrienol-Rich Mixture in Refined Olive Oil and Alpha-Tocopherol on Joint Health in a Rat Model of Osteoarthritis Induced with Monosodium Iodoacetate

**DOI:** 10.3390/ph19101580

**Published:** 2026-10-05

**Authors:** Hiba Murtadha Al-Saadi, Sophia Ogechi Ekeuku, Fairus Ahmad, Nur Vaizura Mohamed, Norliza Muhammad, Kok-Yong Chin

**Affiliations:** 1 Department of Pharmacology, Faculty of Medicine, Universiti Kebangsaan Malaysia, Cheras 56000, Malaysia; hiba.alsaadi2016@yahoo.com (H.M.A.-S.); sogechie@ukm.edu.my (S.O.E.); norliza_ssp@hctm.ukm.edu.my (N.M.); 2 College of Dentistry, Uruk University, Karada 10069, Iraq; 3 Department of Anatomy, Faculty of Medicine, Universiti Kebangsaan Malaysia, Cheras 56000, Malaysia; fairusahmad@ukm.edu.my; 4 Centre for Drug and Herbal Development, Faculty of Pharmacy, Universiti Kebangsaan Malaysia, Kuala Lumpur 50300, Malaysia; vaizura@ukm.edu.my

**Keywords:** arthritis, cartilage, inflammation, joint, vitamin E

## Abstract

**Background/Objectives:** Palm tocotrienol-rich mixture (PT) is a potential disease-modifying agent against osteoarthritis (OA), but direct comparisons with α-tocopherol (AT) are lacking. This study evaluated the joint-protective effects of PT versus AT in a monosodium iodoacetate (MIA)-induced rat model of OA. **Methods:** Male Sprague-Dawley rats (*n* = 6/group) received intra-articular MIA in the left knees to induce OA, followed by daily oral treatment with distilled water (OA control), glucosamine sulphate (250 mg/kg), AT (100 mg/kg), or PT (100 mg/kg, in refined olive oil) for four weeks. Non-OA rats receiving distilled water served as normal controls. Body weight, joint width, and grip strength were monitored weekly. Left knees were harvested for histological evaluation using Mankin scores. Cartilage lysates from the left knees were analysed for inflammatory cytokine and metalloproteinase gene expressions, along with protein phosphorylation ratios of β-catenin (Ctnnb) and nuclear factor kappa-B (Nfkb). **Results:** PT-treated rats showed no significant time-dependent deterioration in joint width or grip strength over the 4-week period. Histologically, PT significantly reduced cartilage structural degradation (*p* = 0.002 vs. OA control) and downregulated *Mmp13* mRNA expression (*p* < 0.05 vs. OA control). Conversely, AT upregulated *Il1b* (*p* = 0.002 vs. OA control; *p* = 0.010 vs. normal control), *Sost* (*p* < 0.05 vs. all OA groups), and *Ctnnb* (*p* = 0.010 vs. OA control) mRNA expressions. However, no intergroup differences in phosphorylation of Ctnnb and Nfkb were observed (*p* > 0.05). **Conclusions:** PT is associated with a chondroprotective profile in MIA-induced OA rats, whereas AT is associated with a catabolic transcriptional pattern. Nevertheless, AT did not cause significant worsening of cartilage structure or activation of pathways under these conditions.

## 1. Introduction

Osteoarthritis (OA) is a progressive, degenerative disease affecting 607 million people globally [1]. It is a leading cause of chronic pain and disability in ageing populations [2]. Characterised by cartilage destruction, subchondral bone remodelling, and low-grade synovitis [3], OA is now understood as a complex inflammatory condition rather than a simple “wear-and-tear” of the joint [4]. Its pathogenesis involves pro-inflammatory cytokines, specifically interleukin (IL)-1β and tumour necrosis factor (TNF)-α, which stimulate matrix-degrading enzymes and promote chondrocyte apoptosis [5], while excessive mechanical loading activates Wnt signalling to further aggravate joint damage [6].

Current pharmacological treatments are primarily palliative, offering transient relief without reversing structural damage [7]. Furthermore, common therapies such as oral non-steroidal anti-inflammatory drugs and corticosteroids carry risks of systemic side effects, including gastrointestinal, renal, and cardiovascular issues, or potential chondrotoxicity [8]. Despite the availability of slow-acting disease-modifying agents, their clinical efficacy remains largely debated [9], highlighting an urgent need for safer treatments that effectively mitigate chronic synovial inflammation.

Vitamin E has gained substantial attention as a potential chondroprotective agent [10]. Vitamin E is broadly divided into tocopherols and tocotrienols, with each group comprising four distinct homologues (α-, β-, γ-, and δ-) [11]. Currently, α-tocopherol (AT) is the most thoroughly investigated homologue in joint diseases due to its high bioavailability and established radical-scavenging capabilities [10]. However, clinical trials and preclinical studies evaluating AT in OA have yielded inconsistent results [12,13]. In contrast, tocotrienol, particularly palm tocotrienol-rich mixture (PT), has emerged as a structurally and functionally superior therapeutic candidate [14]. Structurally, tocopherols possess a saturated phytyl side chain, whereas tocotrienols possess an unsaturated isoprenoid tail containing three double bonds. This structural variance allows tocotrienols to exhibit superior cellular membrane distribution, as well as enhanced antioxidant and anti-inflammatory properties [15].

Various OA models are available to evaluate the therapeutic potential of joint-protective agents. The intra-articular monosodium iodoacetate (MIA)-induced model of OA is one of the most widely utilised [16]. MIA inhibits glyceraldehyde-3-phosphate dehydrogenase, causing glycolytic failure, chondrocyte death, and subsequent cartilage deterioration. Being a stage-progressing model, OA induced by MIA is marked by early synovitis and pain sensitisation (3-7 days post-induction), and late-stage degradation of cartilage structure and subchondral bone remodelling (28 days post-induction) [17]. Thus, the MIA model closely recapitulates the progressive histopathological, biochemical, and nociceptive features of human OA [18].

While PT has demonstrated osteoprotective and anti-inflammatory properties in various disease models [19], a head-to-head comparison of the efficacy of oral PT and AT in mitigating joint degradation in OA has not been established. Therefore, this study aims to compare the structural, biochemical, and behavioural chondroprotective effects of PT versus AT in an MIA-induced rat model of OA. It is hypothesised that due to its superior biological activities, PT would perform better than AT in preserving knee joint function and cartilage architecture while attenuating local inflammation.

## 2. Results

### 2.1. Body Weight, Joint Width, and Grip Strength

**Figure 1:**
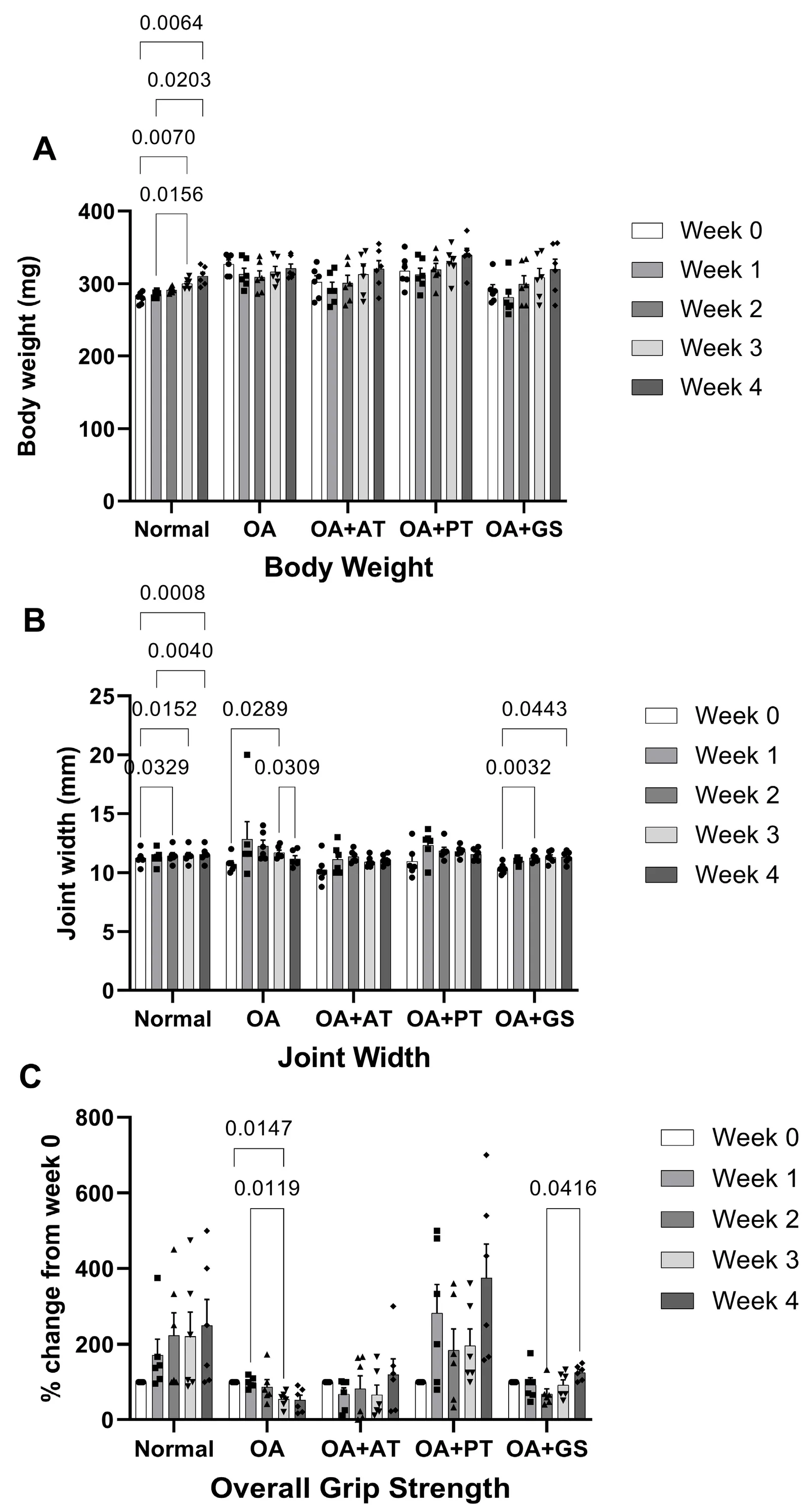
Body weight (**A**), joint width (**B**) and percentage changes in grip strength (**C**) among the study groups (*n* = 6/group). The data are presented as mean ± standard errors of the mean. Mixed-design analysis of variance is used to analyse the time- and group-dependent changes in the parameters. Abbreviation: AT, alpha-tocopherol; GS, glucosamine sulphate; OA, osteoarthritis control; PT, palm tocotrienol-rich mixture. Notes: ●, ■, ▲, ▼ and ◆ represent individual data points for normal, OA, OA+AT, OA+PT and OA+GS groups.

Mixed-design ANOVA revealed significant main effects of time (*p* < 0.001) and treatment (*p* = 0.008) on body weight across the study groups, but the time × treatment interaction effect was not significant (*p* = 0.865). Post hoc analysis showed a time-dependent increase in body weight in the normal control group, but not in the other groups. OA induction and treatments did not affect rat body weight (Figure 1A).

For joint width, a significant time effect (*p* = 0.028) but non-significant treatment effect (*p* = 0.259) was found across the study groups. The time × treatment interaction effect was not significant (*p* = 0.216). Post hoc analysis showed a time-dependent increase in the normal control and OA+glucosamine sulphate (GS) group, and an increase followed by a decrease in the OA group. The OA groups receiving AT and PT did not experience significant time-dependent changes in joint width (Figure 1B).

Regarding grip strength, significant effects of time (*p* = 0.022) and treatment (*p* < 0.001) were observed across the study groups. The time × treatment interaction effect was also significant (*p* = 0.043). Post hoc analysis revealed a time-dependent decline in the OA group and an increase in the OA+GS group. Neither the normal control nor the OA groups receiving AT and PT showed significant time-dependent changes. At the 4th week of treatment, the grip strength of the OA+GS group was significantly higher than that of the OA group (Figure 1C).

### 2.2. Mankin Scores and Circulating Cartilage Oligomeric Matrix Protein (COMP) Levels

**Figure 2:**
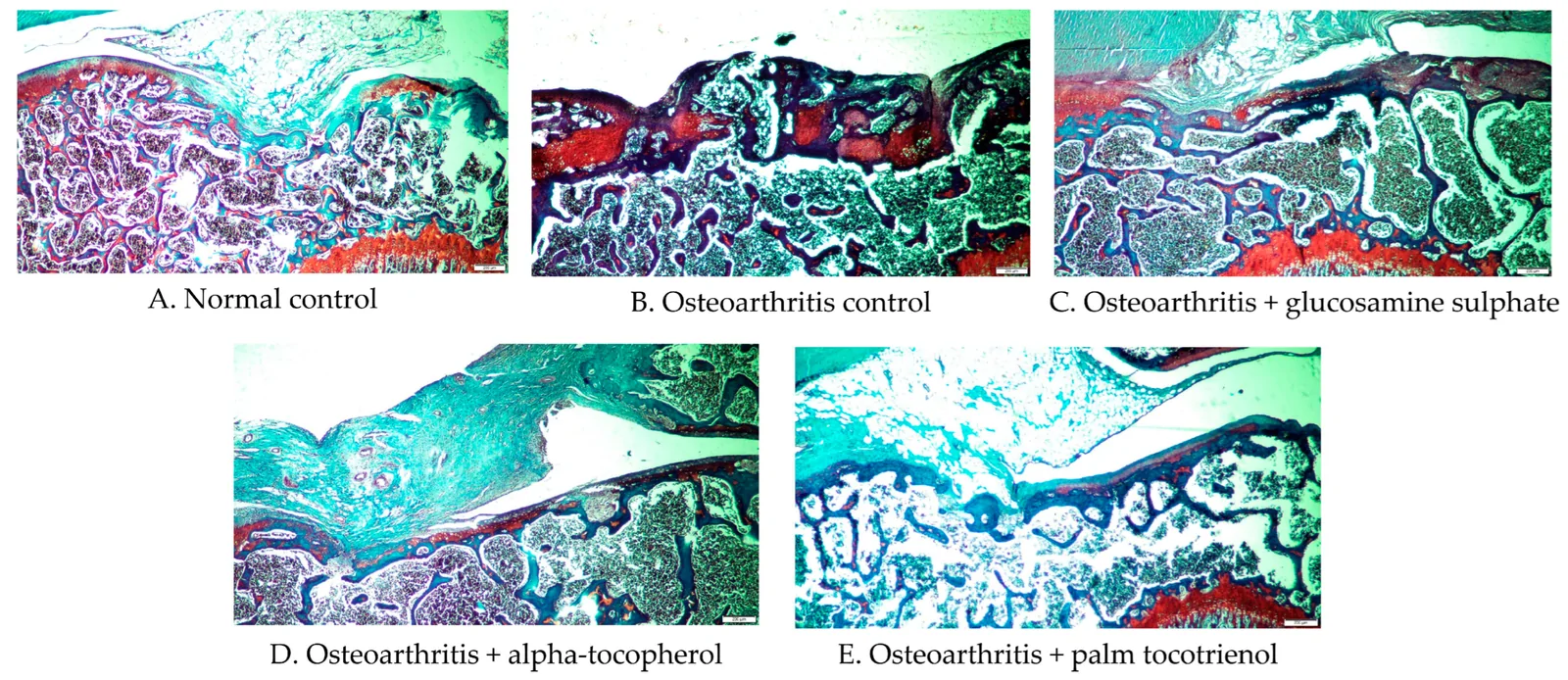
Joint histological features across the study groups, stained using Safranin O and fast green (4× magnification).

**Figure 3:**
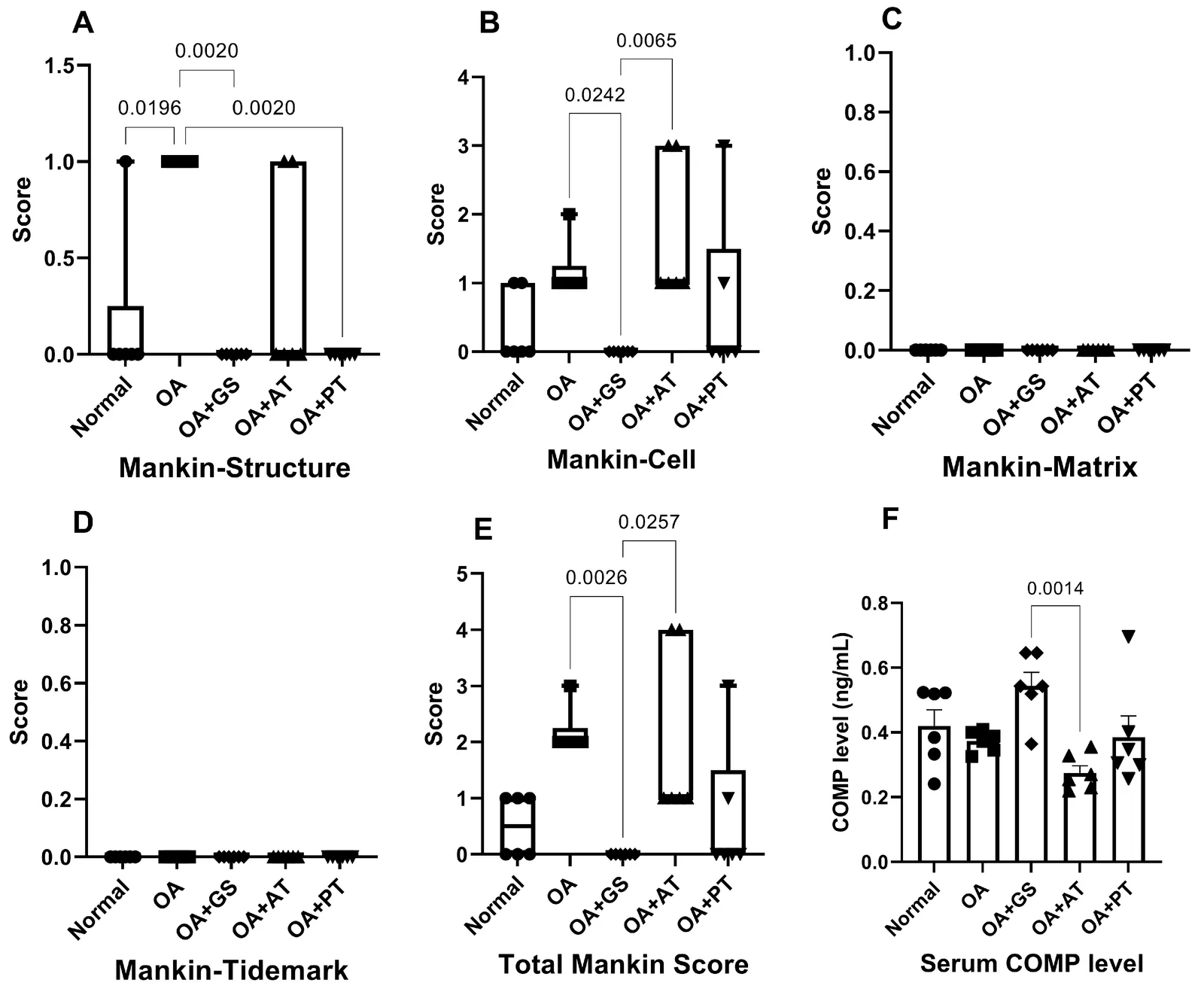
Structure (**A**), cell (**B**), matrix (**C**), tidemark (**D**) and total Mankin scores (**E**), and circulating cartilage oligomeric matrix protein (COMP) (**F**) of the study groups (*n* = 6/group). The Mankin scores are presented as median and interquartile range using box plots. The COMP levels are presented as mean ± standard errors of the mean. Kruskal–Wallis test was used to analyse the differences in Mankin scores. One-way analysis of variance is used to analyse the differences in COMP levels. Abbreviation: AT, alpha-tocopherol; GS, glucosamine sulphate; OA, osteoarthritis control; PT, palm tocotrienol-rich mixture. Notes: ●, ■, ◆, ▲ and ▼ represent individual data points for normal, OA, OA+AT, OA+PT and OA+GS groups.

The joint histological features stained with Safranin O and fast green are presented in Figure 2A–E. The Kruskal–Wallis test revealed a significantly higher structural score in the OA group than in the normal control (*p* = 0.020), but no significant differences in other components or total Mankin scores between the groups (*p* > 0.05). PT significantly reduced the structural score compared with the OA group (*p* = 0.002) but did not alter the other components or the total Mankin score (*p* > 0.05). GS significantly lowered the structural (*p* = 0.002), cellular (*p* = 0.024), and total Mankin scores (*p* = 0.003) compared to the OA group. The OA+AT group showed higher cellular (*p* = 0.007) and total Mankin scores (*p* = 0.026) compared to the OA+GS group (Figure 3A–E).

One-way ANOVA showed that serum COMP levels were significantly higher in the OA+GS group compared to the OA+AT group (*p* = 0.001). No other significant differences were noted across the other study groups (*p* > 0.05) (Figure 3F).

### 2.3. Expression of Genes Related to Inflammation, Cartilage Degradation, and Wnt Signalling in the Cartilage

**Figure 4:**
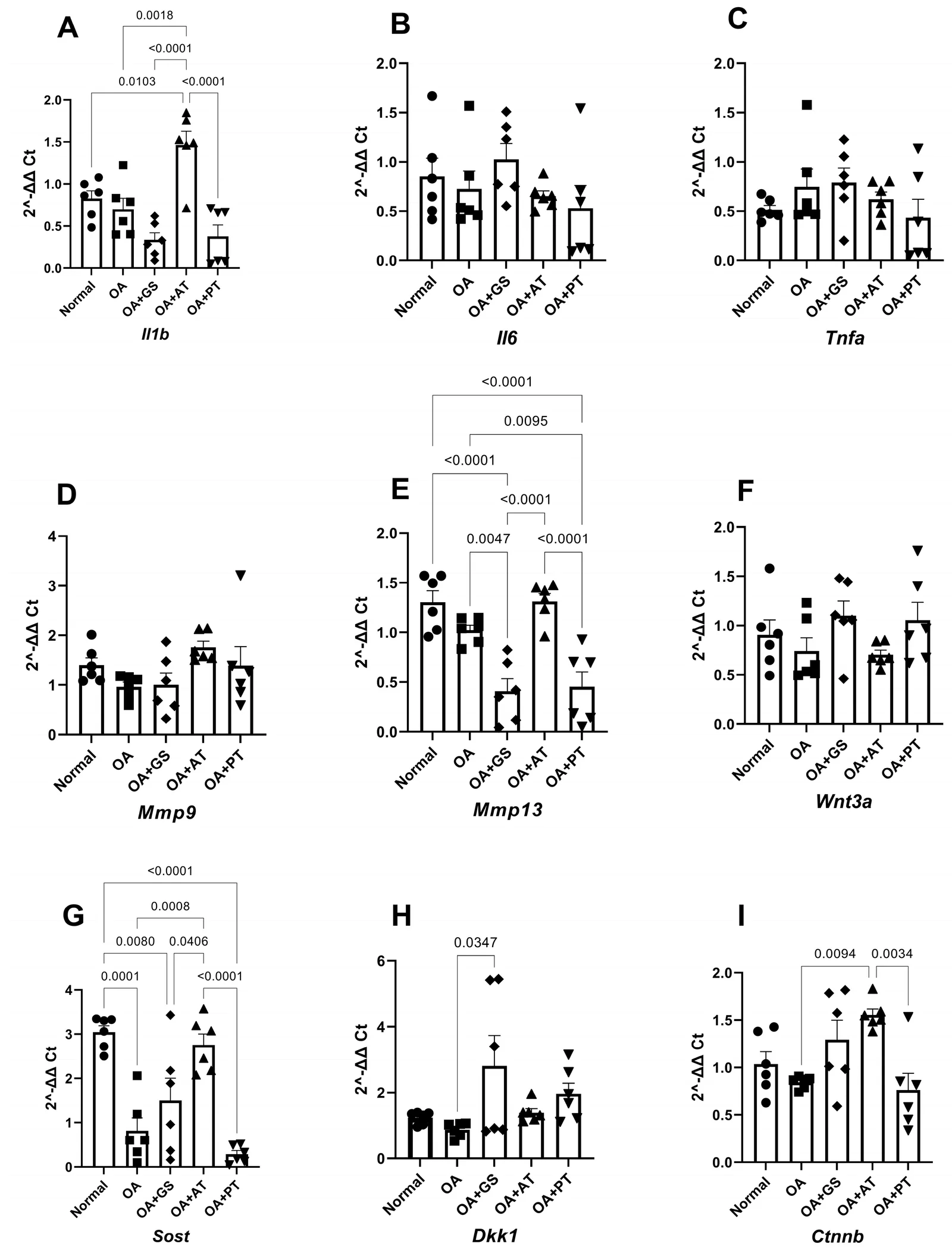
mRNA expression of *Il1b* (**A**), *Il6* (**B**), *Tnfa* (**C**), *Mmp9* (**D**), *Mmp13* (**E**), *Wnt3a* (**F**), *Dkk1* (**G**), *Sost* (**H**) and *Ctnnb* (**I**) among the study groups (*n* = 6/group). The data are presented as mean ± standard errors of the mean. One-way analysis of variance is used to analyse the differences among the parameters. Abbreviation: AT, alpha-tocopherol; GS, glucosamine sulphate; OA, osteoarthritis control; PT, palm tocotrienol-rich mixture. Notes: ●, ■, ◆, ▲ and ▼ represent individual data points for normal, OA, OA+AT, OA+PT and OA+GS groups.

Single-dose MIA induction did not affect *Il1b*, *Il6*, and *Tnfa* expression in the cartilage compared to the normal control after 4 weeks (*p* > 0.05). However, AT increased *Il1b* levels significantly compared with all other groups (*p* < 0.05). PT and GS did not alter the gene expression of these pro-inflammatory cytokines compared to the OA group (*p* > 0.05) (Figure 4A–C).

Similarly, single-dose MIA induction did not affect *Mmp9* and *Mmp13* expression in the cartilage compared to the normal control after 4 weeks (*p* > 0.05). PT (*p* = 0.010) and GS (*p* = 0.005) decreased *Mmp13* expression compared to the OA group. AT increased *Mmp13* expression compared to the OA groups receiving PT (*p* < 0.001) and GS (*p* < 0.001). None of the treatments altered *Mmp9* expression (*p* > 0.05) (Figure 4D–E).

Single-dose MIA induction did not affect *Wnt3a*, *Dkk1* and *Ctnnb* expression in the cartilage (*p* > 0.05) but decreased *Sost* expression (*p* < 0.001) compared to the normal control. AT significantly increased *Sost* expression compared to all other groups (*p* < 0.05), except the normal control (*p* > 0.05). It also increased *Ctnnb* expression compared to the OA (*p* = 0.010) and OA+PT groups (*p* = 0.003). GS increased *Dkk1* levels compared to the OA group (*p* = 0.035). PT did not alter the expression of these markers compared to the OA group (*p* > 0.05) (Figure 4F–I).

### 2.4. NFκB and Ctnnb Phosphorylation in the Cartilage

**Figure 5:**
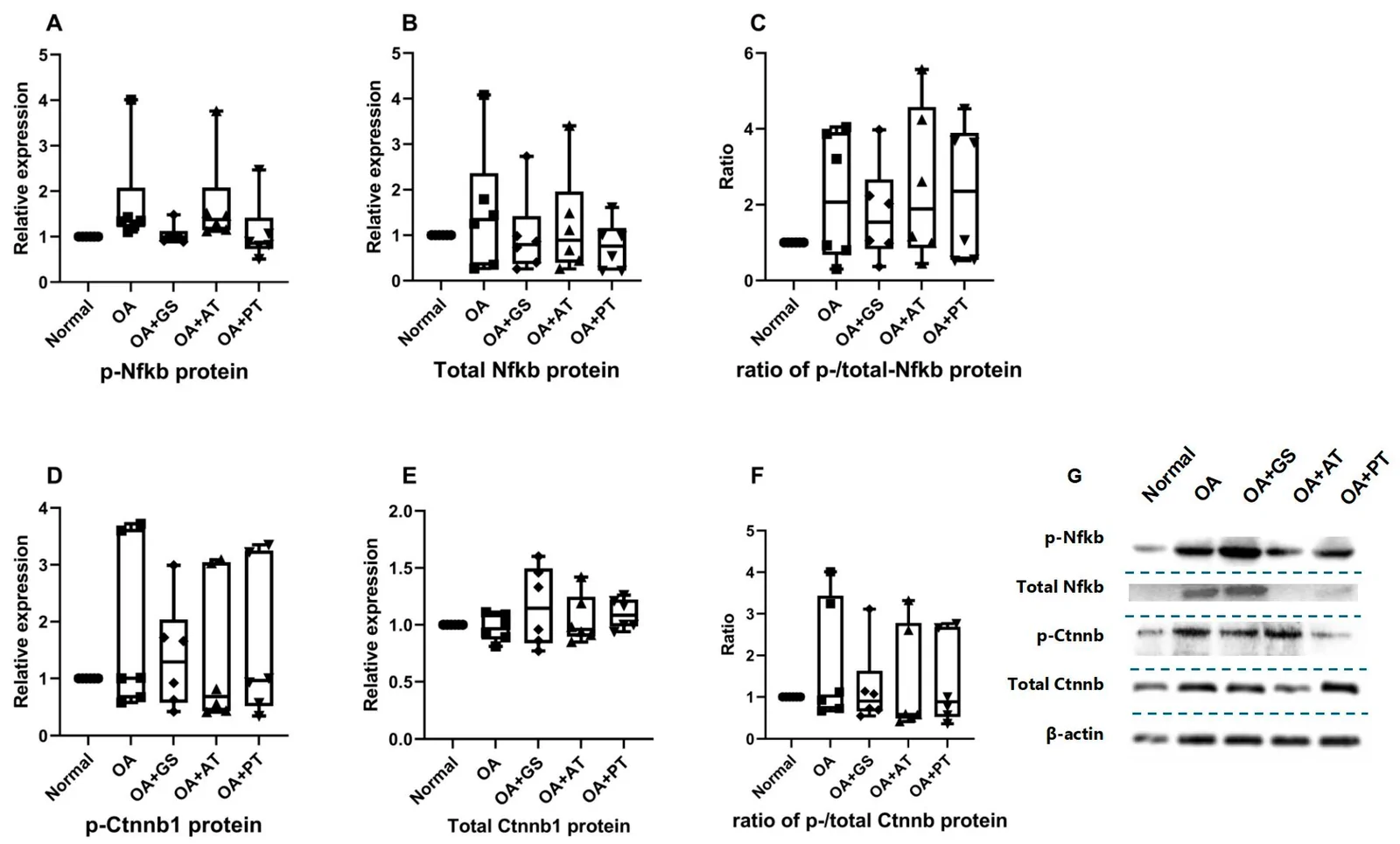
Expression levels of total and phosphorylated Nfkb (**A**,**B**) and Ctnnb (**D**,**E**), along with their respective phosphorylation ratios (**C**,**F**), across the study groups (*n* = 6/group). The protein bands are shown in (**G**), which is a composite assembled from different membranes. The data are presented as median and interquartile range using box plots. Kruskal–Wallis test is used to analyse the differences among the parameters. Abbreviation: AT, alpha-tocopherol; GS, glucosamine sulphate; OA, osteoarthritis control; PT, palm tocotrienol-rich mixture.

Kruskal–Wallis test revealed an omnibus significant difference in phosphorylated Nfkb (*p* = 0.005), but not in total and the ratio of phosphorylated/total Nfkb among the groups (*p* > 0.05). However, Dunn’s post hoc pairwise comparison showed no significant difference in the expression of total, phosphorylated, or the ratio of phosphorylated/total Nfkb was detected in the cartilage across the study groups (*p* > 0.05), likely due to the conservative correction applied in Dunn’s test (Figure 5A–C).

The Kruskal–Wallis test did not reveal any significant difference in total, phosphorylated and the ratio of phosphorylated/total Ctnnb among the groups (Figure 5D–F).

The statistics of all parameters are presented in Table S1.

## 3. Discussion

This study evaluated the therapeutic potential of PT and AT in a rat model of MIA-induced OA, with GS serving as a positive control. The study demonstrated chondroprotective effects of PT, evidenced by reduced cartilage structural damage and suppressed *Mmp13* expression. In contrast, AT led to an unfavourable molecular profile, characterised by upregulated *Il1b* expression and Wnt signalling components. However, significant changes in the phosphorylation of key molecules, Nfkb and Ctnnb, were lacking with both AT and PT supplementation.

Body weight monitoring showed a time-dependent increase in the normal control group, but not in the OA groups. This observation is similar to a previous study, which demonstrated an initial prevention of body weight increase in rats with OA, speculated to be a pain-induced reduction in food intake [20]. No overt treatment-related weight loss was observed.

The normal control demonstrated a gradual but significant increase in knee joint width throughout the experiment, which mirrored its body weight, suggesting normal growth in the rats. The knee joint width in rats with OA showed an initial increase, followed by a decrease, suggestive of early inflammation that gradually subsided. This observation is similar to previous OA models using MIA [17,21]. This is supported by the lack of Nfkb phosphorylation and the absence of pro-inflammatory cytokine elevation in the OA group at week 4. PT- and AT-supplemented rats exhibited changes similar to those in the OA control, but these changes were not significant, indicating partial attenuation of inflammation. However, because PT was administered in a refined olive oil vehicle while the OA groups received distilled water, these initial trends must be interpreted with caution, as vehicle-related fatty acid contributions cannot be completely ruled out. Changes in knee joint width in GS-supplemented rats were similar to those in normal controls, suggesting complete inhibition of inflammation. The effects of GS in preventing joint swelling have been reported in other studies [22]. Evidence on the effects of AT on joint swelling is heterogeneous. In rats with rheumatoid arthritis (RA), AT supplementation (100 mg/kg, i.p., 18 days) reduced paw oedema [23], but its effects were less promising in reducing the number of swollen knee joints in human subjects with RA (1200 mg/day for 12 days) [24].

Grip strength, measured by retention time on the wire mesh, was used to reflect the joint function of the rats [25]. The normal control group showed a gradual, non-significant increase in grip strength. Similar to previous studies [20,21], the OA control group experienced a significant decline in grip strength over time with no sign of rebound. GS-supplemented rats showed an initial decline in grip strength, followed by a significant rebound, indicating improved joint function. In contrast, rats receiving PT maintained stable grip strength throughout the 4-week treatment period, with no significant temporal decline. However, since the post hoc comparisons at week 4 did not reach statistical significance between the PT and OA control groups (unlike the GS group), PT should be interpreted as stabilising functional performance over time rather than actively enhancing joint strength under the present experimental conditions. The grip strength showed individual variability, which could be contributed to by other factors, such as whole-body muscle endurance, motivation and temperament in the rats [26,27], despite our best efforts at optimisation.

Joint histology scoring confirmed the presence of OA in the negative control group, particularly in cartilage structure. Although a tidemark breach is a sign of progressive OA [28], it was not detected in the current study. GS showed the best performance in attenuating adverse joint changes, whereas PT only improved structural changes. This observation is similar to the findings of a previous report [20,21]. In contrast, total Mankin scores in the AT-treated group were significantly higher than those in the GS-treated group, but not significantly different from those in the normal control group. This finding contradicts many previous studies, which demonstrated the protective effects of AT on joint health in OA models [29,30]. However, these studies used higher doses, administered intra-articular injections and different animal models. It should be noted that the Safranin O and fast green staining was darker than expected, which could affect the interpretation of the histological slides.

The structural changes caused by AT supplementation were substantiated by alterations in molecular markers. AT was associated with a significant upregulation of *Il1b* and *Mmp13* mRNA expression, suggesting an inflammation-driven joint-degradation process. However, the exact mechanism underlying this elevation remains unclear, as no significant Nfkb phosphorylation was observed. The findings contradict the reported anti-inflammatory effects of AT in animals and patients with OA [30,31]. For example, AT supplementation (600 mg/kg 3 times per week for 8 weeks) has been reported to reduce serum TNFα and IL6 levels in rats with OA [32]. PT and GS significantly reduced *Mmp13* mRNA expression compared to the OA control, suggesting inhibition of cartilage degradation. These observations align with the reported MMP-inhibiting effects of these agents. In particular, the suppressive effects of GS have been widely reported in many studies [33,34]. However, it is important to note that without a dedicated olive oil vehicle control group, the observed structural preservation associated with PT supplementation cannot be unequivocally attributed to the tocotrienol components, but rather to the PT-olive oil formulation as a whole.

Circulating COMP is conventionally evaluated as a marker of cartilage matrix turnover. In the present study, the only statistically significant difference observed was lower serum COMP levels in the AT-treated group compared with the GS-treated group, with no significant differences detected relative to the untreated OA control or normal groups. Since serum COMP levels were not significantly elevated in AT-treated animals compared with controls, these data do not independently support a detrimental or catabolic effect of AT on cartilage matrix integrity under the present experimental conditions.

Activation of the Wnt signalling pathway is associated with chondrocyte death, ECM degradation and osteophyte formation [35,36]. A concurrent increase in *Sost* and *Wnt3a* mRNA expressions was observed with AT supplementation. It is proposed that the increased *Sost* expression is a negative feedback response to the increase in *Wnt3a*, since *Sost* is a negative regulator of the Wnt pathway [37]. However, the evidence of downstream Wnt pathway activation was lacking. GS increased *Dkk1* mRNA expression, another Wnt pathway inhibitor, but did not affect Ctnnb phosphorylation levels. The lack of change in Ctnnb phosphorylation rate, similar to that of Nfkb, could indicate that transient activation had subsided by week 4.

Overall, the actions of PT and AT on joint health appear to be opposite. PT is associated with joint protection by downregulating *Mmp13*, whereas AT is associated with more severe damage by upregulating joint catabolic pathways. However, the exact mechanism underlying the distinct actions of PT and AT remains unclear. We propose that the higher bioavailability of AT acetate in the body may contribute to pro-oxidant effects and more severe damage to knee joint cartilage, but redox assessments of the joints were not planned to support this speculation. In the Multicenter Osteoarthritis Study, high circulating AT levels have been associated with incident whole-knee radiographic OA [38]. Compared with PT, GS performed better in joint-protective activities. A previous study has already demonstrated that the combination of PT and GS performed better than individual treatments against OA [20]. These findings indicate that PT could act as an adjuvant to GS in combating OA.

When compared directly, GS demonstrated broader histological protection than PT, significantly improving the structural, cellular, and total Mankin scores, whereas PT improved only the structural subscale. At the molecular level, PT was associated with a less catabolic transcriptional profile than AT but did not match the overall efficacy of GS under the present experimental conditions. Nevertheless, GS is contraindicated in patients with shellfish allergy [39], and its long-term gastrointestinal tolerability is a concern in some patients [40]. In this context, PT warrants further investigation as a potential adjunct or alternative in patients for whom GS is not appropriate, provided its efficacy is confirmed in longer-term and higher-powered preclinical models.

Several studies have examined the toxicity aspect of PT. A study on Fischer 344 rats reported the no-observed-adverse-effect level for PT as 120 mg/kg bw/day for male rats and 130 mg/kg bw/day for female rats, based on changes in haematological and liver profile [41]. In another study, 28-day oral supplementation with PT up to 250 mg/kg in female BALB/c mice did not show significant adverse changes in the biochemical profile and liver histology [42]. This dose is equivalent to 107 mg/kg in rats based on body surface area conversion [43]. A recent study showed that exposure to PT below 500 μg/mL in zebrafish embryos caused no significant morphological changes [44,45]. Taken together, these preclinical safety thresholds consistently exceed or align with the 100 mg/kg bw/day dose administered in the current study. Therefore, the oral dose of PT utilised here falls well within the established safety margin for rodent models, ensuring that the observed joint-protective outcomes occurred in the absence of systemic toxicity.

### Limitations

This study has several limitations. The MIA model resembles a progressive, time-sensitive OA model, which presents several issues in the current study. The inflammation and molecular changes had resolved by the end of week 4, complicating the assessment of the agents’ mechanisms of action. Sampling cartilage at this time point missed the early rise in inflammatory markers, thereby contributing to the lack of significant differences between the model and sham groups. Future studies should sample synovial fluid for inflammation at time points that coincide with joint swelling. Furthermore, the current findings should also be validated with chronic OA models induced by surgery, such as anterior cruciate ligament transection, modified medial meniscectomy, or distal meniscal transection [46]. The joint changes observed in this study may represent an acute response to the tested agents, given the relatively short treatment duration (4 weeks). Consequently, these findings may not fully reflect the long-term, chronic outcomes typically associated with therapeutic drug use in patients with OA. Future studies should consider a longer treatment period (8–12 weeks) with an appropriate OA model.

In addition, MIA induction for 4 weeks yielded early-to-moderate structural alterations (as evidenced by a total Mankin’s score of 2–3), reflecting early-stage OA. Evaluating potential disease-modifying agents during early-grade OA is clinically relevant, as early intervention offers the best opportunity to preserve the remaining cartilage architecture before irreversible, end-stage structural failure occurs. However, this approach limits the generalisability of findings to more advanced disease stages. Future studies should employ chronic or higher-dose MIA models, or alternative models, such as destabilisation of the medial meniscus, to determine whether the observed effects of PT are maintained under conditions of greater structural damage.

Since the treatment procedure and grip strength test were not blinded, potential performance bias could not be eliminated. Therefore, the readers should interpret these results with caution. In addition, the inverted wire mesh test reflects whole-body muscular endurance rather than knee joint-specific function. While it has been used as an indirect measure of pain-related functional impairment in rodent OA models [47,48,49], future studies should incorporate joint-specific assessments such as the incapacitance test or gait analysis to characterise joint function more precisely.

The current study examined only the transcriptomic changes in inflammatory cytokines, MMPs, and Wnt signalling inhibitors due to resource constraints. Further validation using protein expression should be performed in future studies. The changes in phosphorylation rates of Ctnnb and Nfkb do not necessarily directly translate to changes in Wnt and NFκB pathway activations. Determination of nuclear Ctnnb or p65 localisation, or the levels of downstream targets, could help confirm the status of these signalling pathways. Complementary in vitro studies using primary chondrocytes and cartilage explants may also validate the findings of this in vivo study.

The AT used in this study was set at the same dose as PT (100 mg/kg) for direct comparison, but this may limit its translational value. Based on body surface area conversion [43], 100 mg/kg used in this study translates to a human equivalent dose of 16.22 mg/kg. For a 60–70 kg human, it is equivalent to a daily dose of 973.2 mg/day to 1135.4 mg/day. Previous human studies investigating the therapeutic effects of oral AT supplementation in patients with OA used a dosage of around 400–500 IU (1 mg synthetic AT = 1.1 IU) [50,51,52]. Therefore, the dose of AT used in the current study was higher compared to previous studies. In addition, the redox status in the joint, though not evaluated in the current study, might explain the difference in action between PT and AT. Refined olive oil was used as the vehicle for PT, but it was not administered to the other groups. Refined olive oil is reported to contain lower concentrations of phytochemicals, but its fatty acid composition (for example, oleic acid) might still have health-enhancing effects that temper the effects of PT. Thus, the effects of PT observed in this study should be interpreted with caution, as solvent effects cannot be entirely ruled out.

Despite these limitations, this study provides the first direct, head-to-head comparison evaluating the efficacy of PT versus AT on joint architecture, functional performance, and molecular pathways within an established MIA-induced OA model. The comprehensive, multi-dimensional methodology ensures a rigorous evaluation of the underlying catabolic and anabolic joint dynamics. These findings offer critical, novel insights into the distinct biological activities of PT versus AT, providing a strong rationale for the development of PT as a potential chondroprotective intervention.

## 4. Methods

### 4.1. Ethical Considerations

The protocol of this study was approved by the Universiti Kebangsaan Malaysia Animal Ethics Committee (approval number: FAR/FP/UKM/2024/CHIN KOK YONG/27-MAC./1419-APR.-2024-MAC.-2026). The conduct of the experiments aligned with the Animal Welfare Act 2015.

### 4.2. Preparation of OA Induction and Treatment Agents

The PT utilised in this study was donated by PhytoGaia Sdn. Bhd. (Kuala Lumpur, Malaysia). It contained 13% AT, 16% α-tocotrienol, 1.2% β-tocotrienol, 18.5% γ-tocotrienol and 4.1% δ-tocotrienol by total content, and the rest was excipient (palm olein) as reported by the manufacturer. The dosage of PT [100 mg/kg body weight (bw)/day] was previously demonstrated to successfully suppress circulating cartilage degradation markers in osteoarthritic rat models [20]. The dosage was calculated based on the total vitamin E content in PT. A single dose of tocotrienol was used because a previous study demonstrated that doses below or above 100 mg/kg bw did not produce better joint protective outcomes [53]. For the current experiment, the agent was diluted in refined olive oil (Bertolli™, Deoleo, Cordoba, Spain) at a 1:5 ratio. Refined olive oil was chosen as the vehicle because it contains a minimal amount of phenolics [54]. AT acetate was obtained from Vitago (Petaling Jaya, Malaysia) and dissolved in distilled water. To establish a benchmark against PT, the AT dosage was maintained at 100 mg/kg bw/day. GS was sourced from Rottapharma Ltd. (Dublin, Ireland) and dissolved in distilled water to achieve a concentration of 10 mg/mL. It was subsequently administered at a dose of 250 mg/kg bw/day, a regimen previously established to yield anti-OA benefits [55]. MIA was acquired from Sigma-Aldrich (St. Louis, MO, USA). Prior to the intra-articular injection, a 4 mg dose of MIA was prepared by dissolving it in 50 μL of normal saline.

### 4.3. Sample Size Calculation

Based on the study by Al-Saadi et al. (2021) [20] and the assumption that PT could normalise the level of circulating COMP in OA rats to that of the normal control, an a priori sample size calculation was performed using G*Power version 3.1.9.7 for Windows (Heinrich-Heine-Universität, Düsseldorf, Germany) with the following inputs: effect size, 1.18; α error probability, 0.05; power, 0.9; and number of groups, 5. The calculation yielded *n* = 4 per group. Two rats were added to each group to account for potential loss.

### 4.4. Treatment of Animals

Thirty male Sprague–Dawley rats (3 months old) were acquired from the Laboratory Animal Resource Unit, Universiti Kebangsaan Malaysia (Kuala Lumpur, Malaysia). The animals were housed under standardised conditions: a 12:12-h light:dark cycle, an ambient temperature of 23 °C, and free access to standard rat chow and tap water. Following a seven-day acclimatisation period, the rats were randomised into five groups (*n* = 6/group). OA was induced via a single intra-articular MIA injection into the left knee joint of four groups. Disease initiation and progression were monitored by measuring the affected joint’s width. Treatments were initiated one day post-OA induction. The normal control and OA control groups were administered distilled water (vehicle control); the positive control group (OA + GS) received GS at 250 mg/kg bw/day; the PT group (OA + PT) was treated with a PT mixture at 100 mg/kg bw/day; and the AT group (OA + AT) received AT acetate at 100 mg/kg bw/day. All treatments were administered daily via oral gavage. Food consumption and body weight were recorded weekly, and blood samples were collected at the end of the treatment period. After four weeks of daily intervention, the rats were euthanised. The left femurs were harvested and preserved in 10% neutral buffered formalin for histological assessment, while the left tibias were collected and stored at −70 °C for molecular analysis.

### 4.5. Randomisation and Blinding

The rats were randomised using a random number generator within a statistical software package (SPSS version 26, IBM, Armonk, NY, USA). Blinding during animal treatment and in vivo assessment (grip strength and joint width) was not performed due to local laboratory constraints. Post-harvest, samples were coded before assessment so that the assessors of the respective parameters were unaware of the animal groupings.

### 4.6. Inverted Wire Mesh Test

Grip strength was evaluated using a modified version of Kondziela’s inverted screen test [25]. The rats were habituated on the mesh screen for 2 min, and they were re-positioned at the centre of a wire mesh screen before the test commenced. Timing was initiated within two seconds of inverting the screen, ensuring the animal’s head was oriented downward first. The screen was secured steadily at a height of 60 cm above a padded surface. The total time until the animal fell was recorded. If the rats did not fall, they were removed from the mesh wire after 120 s. Each rat was given three trials, and the best performance was recorded. This behavioural assessment was conducted prior to the MIA injection and repeated weekly until euthanasia. Due to high baseline variability among the rats, the data were converted to the percentage of change versus week 0 using the following formula:

Percentage change = [(retention time at the respective week − retention time at week 0)/retention time at week 0] × 100%.

### 4.7. Joint Width Measurement

The rats were manually restrained, and the transverse diameter of the left knee joint was measured with a digital caliper placed across the lateral aspects of the joint. These measurements were performed weekly.

### 4.8. Histological Assessment

Soft tissues of the left femurs were removed. Then, the femurs were sectioned into two halves while maintaining the integrity of the articular cartilage. Half of the specimen was decalcified in 10% ethylenediaminetetraacetic acid for 30 days and subsequently embedded in paraffin wax. Histological sections (8 μm) were stained with Safranin O and Fast Green, followed by evaluation by a single blinded anatomist utilising the modified Mankin scoring system (Table 1).

### 4.9. Circulating COMP Levels

Blood samples were collected in plain tubes and centrifuged at 3000 rpm for 10 min at 4 °C to isolate the serum, which was stored at −70 °C until analysis. Serum COMP levels were quantified using an enzyme-linked immunosorbent assay kit (Finetest, Wuhan, China) following the manufacturer’s instructions.

### 4.10. Sample Preparation for Molecular Studies

The articular cartilage layer on the left tibias was scraped using a scalpel, pulverised in liquid nitrogen, and divided into two equal portions. Total RNA was extracted from one portion using TRIzol reagent (Thermo Fisher Scientific, Waltham, MA, USA). Total proteins were extracted from the other portion using RIPA buffer (Beyotime, Shanghai, China) supplemented with protease and phosphatase inhibitors (Thermo Fisher Scientific, Waltham, MA, USA).

### 4.11. Gene Expression Analysis

To assess the transcription of genes associated with inflammation (*Il1b*, *Il6* and *Tnfa*), cartilage breakdown (*Mmp9* and *Mmp13*), and the Wnt pathway (*Ctnnb*, *Dkk1*, *Sost* and *Wnt3a*), real-time quantitative reverse transcription PCR (qRT-PCR) was executed. Total RNA was isolated, and its concentration and optical purity were verified spectrophotometrically at 260 nm using a NanoDrop instrument (Thermo Fisher Scientific, Waltham, MA, USA). For first-strand complementary DNA (cDNA) synthesis, the purified RNA template was reverse-transcribed via the OneScript Plus cDNA synthesis kit (Applied Biological Materials Inc., Vancouver, BC, Canada) under a thermocycler profile of 55 °C for 15 min, followed by a 5-s enzyme inactivation step at 85 °C. Quantitative PCR amplification was conducted in technical duplicates for each sample, in a total volume of 20 μL per reaction, incorporating the synthesized cDNA, BlasTaq 2X qPCR MasterMix (Applied Biological Materials Inc.), forward/reverse primer pairs (Table 2; Integrated DNA Technologies, Coralville, IA, USA), and nuclease-free water (Vivantis Technologies, Shah Alam, Malaysia). This assay was run on an MIC PCR System (Bio Molecular Systems, Queensland, Australia). The thermal cycling conditions comprised an initial 3-min denaturation at 95 °C, followed by 45 cycles of 95 °C for 3 s (denaturation) and 60 °C for 30 s (combined annealing and extension). Following thermal cycling, melting-curve analysis was conducted according to the standard protocol in the MIC PCR Workbench (Bio Molecular Systems, Queensland, Australia). Samples were heated from 72 °C to 95 °C at a rate of 0.3 °C/s. Raw fluorescence signal drops were converted by the software into derivative plots (−dF/dT vs. temperature) to verify single, distinct melting peaks for each target gene and rule out non-specific amplification or primer-dimer formation. The relative expression of each gene was calculated using *Gapdh* and expressed relative to the normal control using the 2^−ΔΔCt^ method.

### 4.12. Western Blot Analysis

Protein yields were first determined via the bicinchoninic acid approach. Equivalent protein masses were separated using 12% sodium dodecyl sulfate-polyacrylamide gels (Elabscience, Wuhan, China), run under a constant voltage regimen of 80 V through the stacking matrix and 120 V across the resolving gel. Each lane of the gel corresponded to one animal used in the experiment, and the samples were not pooled. Pre-stained standards were included for molecular weight reference. Electrotransfer of the resolved proteins onto polyvinylidene difluoride membranes (pre-activated in methanol; PALL Corporation, Port Washington, NY, USA) was executed at a constant 100 V for 120 min. Nonspecific active sites were blocked using a 5% skimmed milk solution prior to an overnight primary antibody incubation at 4 °C. After iterative rinsing steps in Tris-buffered saline with Tween 20, the blots were exposed to secondary antibodies prepared in 2% skimmed milk. Following a final washing cycle, immunoreactive bands were developed using enhanced chemiluminescence reagents. Emitted signals were documented on a ChemiDoc XRS+ system (Bio-Rad, Hercules, CA, USA) with consistent exposure settings across blots to ensure data reproducibility. Band densitometry was subsequently analysed via ImageJ 1.54r software (National Institutes of Health, Bethesda, MD, USA). Complete antibody specifications are catalogued in Table 3. A common internal calibrator or pooled reference lane was not used to assess interblot variation in the current study, which represented a major limitation of the study. The phosphorylation ratio was calculated by dividing the densitometric value of the phosphorylated protein band by that of the corresponding total protein band, with both values independently normalised to β-actin as the loading control.

### 4.13. Statistical Analysis

Data from all rats were included in the analysis. No rats were excluded due to premature death or sickness. Statistical analysis was performed using GraphPad Prism 9.0 (Dotmatics, Boston, MA, USA). Normality of the data was assessed using the Shapiro–Wilk test. Data that displayed a normal distribution were analysed using one-way analysis of variance (ANOVA) followed by Tukey’s post hoc pairwise comparisons. Otherwise, data were analysed using the Kruskal–Wallis test followed by Dunn’s post hoc comparison. For data featuring a time × group design, a mixed-design analysis of variance was performed to assess the time and treatment effects. A *p*-value < 0.05 was interpreted as establishing statistical significance.

## 5. Conclusions

PT and AT exert differential effects on joint health. Under the present experimental conditions and dosage, PT demonstrates partial protective effects against OA, while AT is associated with a more catabolic molecular profile, although clear evidence of signalling activation or structural worsening has not been demonstrated. As PT was delivered in refined olive oil, the potential confounding contribution of the vehicle should be taken into account when interpreting these outcomes. Given the limitations of this study, the conclusion should be interpreted with caution, as the tissue sampling frame for inflammatory markers may not be optimal. Besides, the effects of GS are superior to those of PT in many domains. Therefore, these findings should not be interpreted as supporting immediate clinical replacement of conventional therapies. Instead, this study provides a rationale for further preclinical investigation of PT, including longer-term chronic OA models and combined regimens with GS, to fully characterise its potential prior to patient-oriented translational positioning.

## Figures and Tables

**Table 1:** The Mankin scoring system for joint histology.

Score	Structure	Cellularity	Matrix Staining	Tidemark Integrity
0	Smooth surface/normal	Normal arrangement	Normal staining	Normal and intact
1	Roughened surface/single crack or area of delamination	Clustering in the superficial layer or loss of cells up to 10%	Slight loss of stain	Disrupted
2	Multiple cracks/moderate delamination	Disorganisation or loss up to 25%	Moderate loss of stain	n/a
3	Fragmentation in cartilage or severe delamination	Cell rows are absent or loss up to 50%	Severe loss of stain	n/a
4	Loss of fragments	Very few cells present	No stain presents	n/a
5	Complete erosion of the tidemark	n/a	n/a	n/a
6	Erosion beyond the tidemark	n/a	n/a	n/a

**Table 2:** The primer sequences used in qRT-PCR.

Target Gene	Forward Primer (5′–3′)	Reverse Primer (5′–3′)
*Mmp9*	CCATGGATCCAGGTTACCCCACT	TGTGGTCCACTGAAGAAGAGGAAGA
*Mmp13*	CACTATGATTGCCGAGAA	AACACGCTTGCTATGCTA
*Il1b*	GGATGATGACCTGCTAGT	CACTTGTTGGCTTATGTTCTGTC
*Il6*	CCAACTTCCAATGCTCTCCTAAT	CGAGTAGACCTCATAGTGACCTT
*Tnfα*	ATGTGGAACTGGCAGAGGAG	AGTAGACAGAAGAGCGTGGTG
*Ctnnb*	ACAGCACCTTCAGCACTCT	AAGTTCTTGGCTATTACGACA
*Wnt3a*	TGCAAATGCCACGGACTATC	AGACTCTCGGTGTTTCTCTACC
*Dkk1*	ATGAGGCACGCTATGTGCTG	CTCGAGGTAAATGGCTGTGGTC
*Sost*	TGCTTCGGTGACAGGTAG	CACGTCTTTGGTGTCATAAG
*Gapdh*	GCAAGAGAGAGGCCCTCAG	TGTGAGGGAGATGCTCAGTG

**Table 3:** Details of the antibodies used in Western Blot procedures.

Target	Host	Dilution	Size (kDA)	Catalogue	Brand
NF-κB (p65/RelA)	Rabbit	1:1000	65	A19653	Abclonal, Woburn, MA, USA
p-Nfkb (p-Ser311 of p65)	Rabbit	1:1000	65	GTX32255	GeneTex, Irvine, CA, USA
Ctnnb	Mouse	1:1000	92	E-AB-70230	Elabscience, Wuhan, China
p-Ctnnb (p-Ser37)	Rabbit	1:1000	85	E-AB-20828	Elabscience, Wuhan, China
Actb	mouse	1:5000	40	E-AB-48018	Elabscience, Wuhan, China

## Data Availability

The data presented in this study are available on request from the corresponding author due to institutional restriction in patent filing.

## Supplementary Materials

The following supporting information can be downloaded at: https://www.mdpi.com/article/10.3390/ph19101580/s1, Table S1: The Statistics of all parameters.
