# Supplementary material for: Divergent Effects of Palm Tocotrienol-Rich Mixture in Refined Olive Oil and Alpha-Tocopherol on Joint Health in a Rat Model of Osteoarthritis Induced with Monosodium Iodoacetate
Source: Pharmaceuticals (Basel). 2026 Oct 5;19(10):1580. doi: 10.3390/ph19101580 (PMC13708357; doi:10.3390/ph19101580)
Supplement: Supplementary file 1 [file pharmaceuticals-19-01580-s001.zip › pharmaceuticals-4548459-supplementary.pdf]

Table S1. The statistics of all parameters

Parameter	Test Type	Statistic	DF	P-value	Effect Size
Body Weight					
Treatment	Mixed-design ANOVA	F=4.398	(4, 25)	0.0079	0.413
Time		F=11.13	(1.83, 45.89)	0.0002	0.308
Treat x Time		F=0.615	(16, 100)	0.8651	0.0896
Joint Width	Mixed-design ANOVA				
Treatment		F=1.411	(4, 25)	0.2592	0.1842
Time		F=7.888	(1.55, 38.71)	0.0028	0.2398
Treat x Time		F=1.294	(16, 100)	0.2161	0.1715
Grip Strength	Mixed-design ANOVA				
Treatment		F=15.09	(4, 25)	<0.0001	0.3765
Time		F=3.477	(1.95, 48.73)	0.0217	0.3574
Treat x Time		F=1.787	(16, 100)	0.0433	0.2223
Mankin Score					
Structure	Kruskal–Wallis	H=19.03	4	0.0008	0.6562
Cell	Kruskal–Wallis	H=16.79	4	0.0021	0.579
Total	Kruskal–Wallis	H=18.37	4	0.001	0.6334
Serum COMP	One-way ANOVA	F=5.147	4, 25	0.0036	0.4515
Gene Expression					
Il1b	one-way ANOVA	F=13.06	(4, 25)	<0.0001	0.6822
Il6	one-way ANOVA	F=1.20	(4, 25)	0.3364	0.1637
Tnfa	one-way ANOVA	F=1.19	(4, 25)	0.34	0.1601
Mmp9	one-way ANOVA	F=2.17	(4, 25)	0.103	0.2575
Mmp13	one-way ANOVA	F=16.37	(4, 25)	<0.0001	0.7232
Wnt3a	one-way ANOVA	F=1.62	(4, 25)	0.1994	0.2059
Sost	one-way ANOVA	F=16.63	(4, 25)	<0.0001	0.7275
Dkk1	one-way ANOVA	F=2.93	(4, 25)	0.041	0.319
Ctnnb	one-way ANOVA	F=5.69	(4, 25)	0.0021	0.4762
Western Blot					
p-NFKB	Kruskal–Wallis	H=14.67	4	0.0054*	0.5059
Total Nfkb	Kruskal–Wallis	H=3.02	4	0.5545	0.1041
Ratio p-/total	Kruskal–Wallis	H=1.50	4	0.8274	0.0516
p-Ctnnb	Kruskal–Wallis	H=1.901	4	0.7539	0.0656
Total-Ctnnb	Kruskal–Wallis	H=2.412	4	0.6604	0.0832
Ratio p-/Total	Kruskal–Wallis	H=2.051	4	0.7263	0.0707

* No post hoc significance was detected with Dunn's multiple comparisons.
